# Chemical Composition Analysis of *Atropa belladonna* Grown in Iran and Evaluation of Antibacterial Properties of Extract-loaded Nanofibers

**DOI:** 10.5812/ijpr-137839

**Published:** 2023-08-06

**Authors:** Elmira Danaie, Shiva Masoudi, Nasrin Masnabadi

**Affiliations:** 1 Department of Chemistry, Central Tehran Branch, Islamic Azad University, Tehran, Iran; 2 Department of Chemistry, Roudehen Branch, Islamic Azad University, Roudehen, Iran

**Keywords:** *Atropa belladonna*, HPTLC, HPLC, GC-MS, Nanofibers, Antioxidant, Antibacterial

## Abstract

In the present work, the chemical composition of extract fractions of the *Atropa belladonna* plant growing in the north of Iran was investigated by HPTLC, HPLC, and GC-MS. Based on HPLC results, atropine, and scopolamine were found to be higher in the fruit and leaf extracts than in other parts of the plant. The comparative GC-MS analysis showed that diacetone alcohol, mesityl oxide, palmitic acid, and linoleic acid were the major bioactive components in the root, stem, leaf, and fruit extracts, respectively. Leaf extract showed the best antioxidant activity in the DPPH test. The antibacterial activity of fractional extracts was determined against *Pseudomonas aeruginosa* using the MIC method, and the fruit and leaf extracts exhibited the best antibacterial activities. The leaf extract was embedded into nanofibers by electrospinning technique, and its antibacterial activity was determined against *Pseudomonas aeruginosa*. The morphology and mechanical properties of the nanofibers were studied with SEM, contact angle, and tensile analysis, showing ultrafine fibers with uniform morphology.

## 1. Background

Infectious diseases are the leading cause of early death worldwide. According to a report by the Centers for Disease Control and Prevention (CDC), more than 2.8 million antibiotic-resistant infections occur yearly in the United States (1). Therefore, there is a need to search for new effective compounds against pathogenic bacteria and fungi. Plant-derived components are promising options for developing new antimicrobial agents. These natural compounds have advantages compared to synthetic alternatives, such as availability in local communities, low price, and ease of administration. Moreover, synthesizing these bioactive structures on an industrial scale involves a very high cost, and, in most cases, it is not feasible because of their complicated structures with several chiral centers. Thus, plant-derived antimicrobial compounds have attracted the attention of many researchers.

*Atropa belladonna* belongs to the Solanaceae family and is native to Europe, North Africa, and Western Asia. This plant also grows in north Iran, locally known as *Shaahbizak*. Up to 20 alkaloids have been found in fruits, leave, and roots of *Atropa belladonna*; the two most important are atropine and scopolamine, which are primarily responsible for the anticholinergic properties of *Atropa belladonna*. Despite the neurotoxic effect of *Atropa belladonna*, several studies have reported its therapeutic effects on inflammation, pain, and infection (2). The presence of bioactive alkaloids is the main reason for their antimicrobial properties (3).

Nanofibers from plant extract provide certain advantages over instant plant extract. First, nanofibers have a larger surface area than traditional plant extracts, allowing for more effective delivery of the antibacterial agent. Additionally, nanofibers can be engineered to release the plant extract slowly over time, prolonging the antibacterial effect. Furthermore, nanofibers can allow targeted delivery of the antibacterial agent to specific areas, reducing the risk of side effects or damage to healthy tissues. Finally, nanofibers can be easily incorporated into various materials, such as wound dressings or surgical implants, making them a versatile and effective tool in the fight against bacterial infections (4, 5).

## 2. Objectives

This study obtained ethanolic extract from the separate parts of the plant, including fruit, leaf, stem, and root. The components were identified using high-performance thin-layer chromatography (HPTLC), and a comparative quantitative analysis of alkaloids and other components in fractional extracts was performed by high-performance liquid chromatography (HPLC) and Gas Chromatography-Mass Spectrometry (GC-MS). An antibacterial mat was prepared via the electrospinning method from leaf extract. The antibacterial activities of fractional extracts and nanofibers obtained from *Atropa belladonna* were evaluated against the gram-negative bacterium *Pseudomonas aeruginosa* to find effective and safe antimicrobial agents with less chance of antibacterial resistance.

## 3. Methods

### 3.1. Materials and Instruments

All chemical compounds, solvents, and cultural media were supplied by Merck Company (Merck, Darmstadt, Germany). The HPTLC detection was carried out with a CAMAG^®^ TLC device, and HPLC-UV was conducted in the Knauer chromatographic system. The GC-MS analysis was performed by MS 5973, GC 6890, Agilent, USA. Nanofibers were obtained from a full-option electrospinning machine from Nano Azma Company, Iran. Also, a scanning electron microscope (SEM) was recorded by a MIRA3 SEM device, TESCAN Company, Czech Republic. The contact angle test was performed with the drop shape analyzer, OCA 15 Plus, Data Physics, Germany. The mechanical properties of nanofibers were investigated by the tensile strength device, SANTAM, STM-20, Iran. Ultraviolet-visible measurements were conducted using a Human crop XMA-2000 UV/Vis spectrophotometer.

### 3.2. Plant Collection and Extraction of Plant Material

*Atropa belladonna*, known locally as *Shaahbizak*, was collected from the Shafarood region, Guilan, Iran. Various parts of the plant, including root, stem, leaf, and fruit, were separated and shade-dried at room temperature. Each part was separately extracted via the Eapen et al. method (6) with slight modifications. Briefly, a specific weight was taken from each part of the dried plant, crushed, and immersed in methanol for 48 hours. Then, the mixture of plant/methanol was filtered several times and put on a bain-marie to obtain the concentrated extract. The root, leaf, and stem of the *Atropa belladonna* plant were fractionated. To extract fatty acids and other organic acids, for each gram of the mixed methanol extract, a mixture of 4 mL of water and 1 mL of sulfuric acid was added to the desired extract, and then extraction was carried out with chloroform (2 × 30 mL). In order to extract alkaloids and other amine components from the remaining methanol extract, 4 mL of water was added, and after pH adjustment at 10 by ammonia, extraction with chloroform (2 × 30 mL) was performed. Organic phases from two steps of extractions were combined, and chloroform was evaporated under reduced pressure at room temperature.

### 3.3. HPTLC

To perform the HPTLC test on *Atropa belladonna* plant fractions, the extracts of different parts of the plant were dissolved in methanol. The dried TLC was placed in the tank containing the appropriate mobile phase (7). The derivatization agents were utilized for better resolution and identification. Rutin, quercetin, benzoic acid, gallic acid, and warfarin were used to identify specific flavonoids. Belladonna, atropine, phenobarbital, and hyoscine were employed to recognize alkaloids. The characteristics of stationary and mobile phases and derivatization agents are given in Appendix 5 in the Supplementary File.

### 3.4. HPLC

The HPLC analysis was performed to quantify the identified alkaloids in extracts. Standard solutions of 0.1 mg/mL were prepared by dissolving atropine and scopolamine standard powders in methanol. The dried extracts were mixed with 5 mL methanol to obtain a sample solution, which was then sonicated for 20 minutes to dissolve the solids completely. The HPLC method was based on Hosseini et al. study (8). In this method, chromatographic separation was performed on a C18 Intersil ODS-4 (4.6 mm × 250 mm, 5 µM) analytical column. The column temperature was fixed to 35°C. The elution isocratic was created from mobile phase Acetonitrile/KH_2_PO_4_ 50 mM (20:80). The solvent flow rate was kept at 1.5 mL min^-1^, and the injection volume was set to 20 µL.

### 3.5. Gas Chromatography-Mass Spectrometry

To measure the volatile components in the extracts, GC-MS analysis was performed. An HP5-MS 30 m × 250-micron capillary column was used to separate volatile compounds. The temperature was maintained at 120°C, then increased to 220°C over 30 min (9). Samples were prepared by adding 5 mL methanol to the dried extract. One microliter of sample solutions was injected into the GC column. A NIST library (version 14) was used to identify each peak according to their MS spectra.

### 3.6. Production of Nanofibers by Electrospinning Method

The method, polymer, solvent, and test conditions are very important in the formation and quality of produced nanofibers. Therefore, after several investigations, it was found that the electrospinning of the desired extract should be done by the double-sided and rotating nozzle method (10). In this method, the Polycaprolactone Polymer (PCL) solution was discharged from a nozzle and collected on a rotating collector. This part would be a body or a scaffold for the solution containing the extract, polyethylene oxide (PEO) polymer, and chitosan, which is placed on the rotating collector from the other side by another nozzle. The leaf has a larger amount of the total extract than the other plant parts. Hence, *Atropa belladonna* leaf extract was used for the electrospinning tests.

The manufacturing process included two steps. In the first step, a 2.5% w/v chitosan and 5% w/v PEO solution was prepared using 5 mL of distilled water after stirring overnight in the presence of a 1% w/v of acetic acid. After the complete dissolution of the two substances, PEO and chitosan, at a ratio of 80:20, were mixed and allowed to be completely dissolved. Then, from the mixture of the two substances, 5 mL was kept as the blank, and the desired extract was dissolved in another 5 mL. In the second step, a 20% w/v of PCL was dissolved in a mixture of acetic acid and formic acid at a ratio of 40:60 and left overnight; this part would not contain any other substance. This part was also prepared in 10 mL; half was used as the blank, and the other half was employed to load 1 wt% of the leaf extract. Two syringes with 23 G (chitosan-PEO solution, PCL solution) were connected to a high voltage of 15.5 KV with a flow rate of 0.3 mL/h for PEO/Chitosan and 0.1 mL/h for PCL. The distance between each needle and collector was 15 cm. The produced nanofiber was collected on a sheet of flat aluminum foil.

### 3.7. Scanning Electron Microscope (SEM) Analysis

The morphology of the obtained nanofibers was examined using the SEM instrument MIRA3, TESCAN Company. For this, ultrathin films of each formulation of nanofibers were prepared to study the surface morphology.

#### 3.7.1. Contact Angle Test

The surface hydrophobicity of nanofibers was investigated by contact angle test using a drop shape analyzer, OCA 15 Plus. The droplet used was distilled water and was 1 µL in volume. The contact angle experiments were carried out at room temperature within 20 s of placement of the water droplet on the electrospun fiber mat.

#### 3.7.2. Tensile Test

For the tensile test of the nanofibers, SANTAM, STM-20, was used. The top of the mat was fixed tightly by the device holder, and the bottom was pulled down by the movement of the other holder so as to elongate the nanofibers.

#### 3.7.3. Antioxidant Test

Due to the presence of many natural compounds in the *Atropa belladonna* plant extract, it was estimated with antioxidant properties. As a result, the antioxidant test was performed with the 1,1-diphenyl-2-picrylhydrazyl (DPPH) method (11). First, a solution of DPPH in methanol was prepared (0.135 mM, 0.0053 g/100 mL). Then, 1.5 mL of different concentrations of the extract in methanol (1000, 500, 250, 125, 62.5, 31.25, 15.625, 7.813, 3.906, 1.953 µg/mL) were added to 1.5 mL of DPPH solution in the test tube. Next, 1.5 mL of methanol was used as a control solution. Ascorbic acid was used as a standard substance at concentrations of 10, 5, 2.5, 1.25, and 0.625 mg/mL in water. Ascorbic acid was added to the DPPH solution. Three repetitions were considered for each concentration. The extract control was also used to eliminate the absorbance of the extract. In the end, the absorption of the solutions was measured at a wavelength of 517 nm after keeping the mixtures for 30 minutes in the dark. The percentage of inhibition was plotted against concentration, and the IC_50_ was calculated from the graph (12, 13).

### 3.8. Microorganisms

The test microorganisms included the ISO standards and clinical isolation of *Pseudomonas aeruginosa*. The *Pseudomonas aeruginosa* (ATCC 9027) standard strain was obtained from the Iranian Research Organization for Science & Technology provided by the American Type Culture Collection (ATCC). Clinical isolates of this microorganism were obtained from hospitalized patients in Motahary Burns Hospital, Tehran, Iran. Gram staining, catalase, oxidase, and arginine dihydrolase tests were performed to identify the *Pseudomonas* strains.

### 3.9. Determination of MIC and MBC

According to the CLSI standard, the Mueller-Hinton broth culture medium was used. The micro broth dilution method was utilized to evaluate the susceptibility of *Pseudomonas* strains to fractional extracts. Each microplate well contained 100 microliters of broth medium; next, 100 microliters of each extract were added to the first chamber, and then 100 microliters were removed and poured into the second chamber. This dilution process continued until well number 11. According to the standard, after making a microbial suspension with a concentration of 0.5 McFarland, 1 mL of this suspension was diluted in 1 mL of Mueller-Hinton broth culture medium, and then 10 microliters of this suspension were poured into each well. The last row was considered a negative control, and no bacterial suspension was added to it. The microplates were kept in an incubator for 24 hours at 37°C.

The MBC test indicates the minimum required concentration of the extract lethal to the microorganism. To perform this test, a Mueller-Hinton agar culture medium was needed. Twenty microliters of the solutions inside the well, where the MIC was observed, were poured on the agar medium's surface. The plates were again placed in an incubator at 37°C for 24 h.

The disk diffusion test was used to evaluate extract-loaded nanofibers' antimicrobial efficacy. Gentamicin was used as a positive control since it is a broad-spectrum antibiotic. First, an 10^8^ CFU/mL suspension was prepared according to 0.5 McFarland and cultivated on Mueller-Hinton agar medium. The nanofibers, with and without extract loading, were then added to the medium and incubated at 37°C for 24 hours.

## 4. Results and Discussions

### 4.1. Qualitative HPTLC Analysis

**Figure 1.**
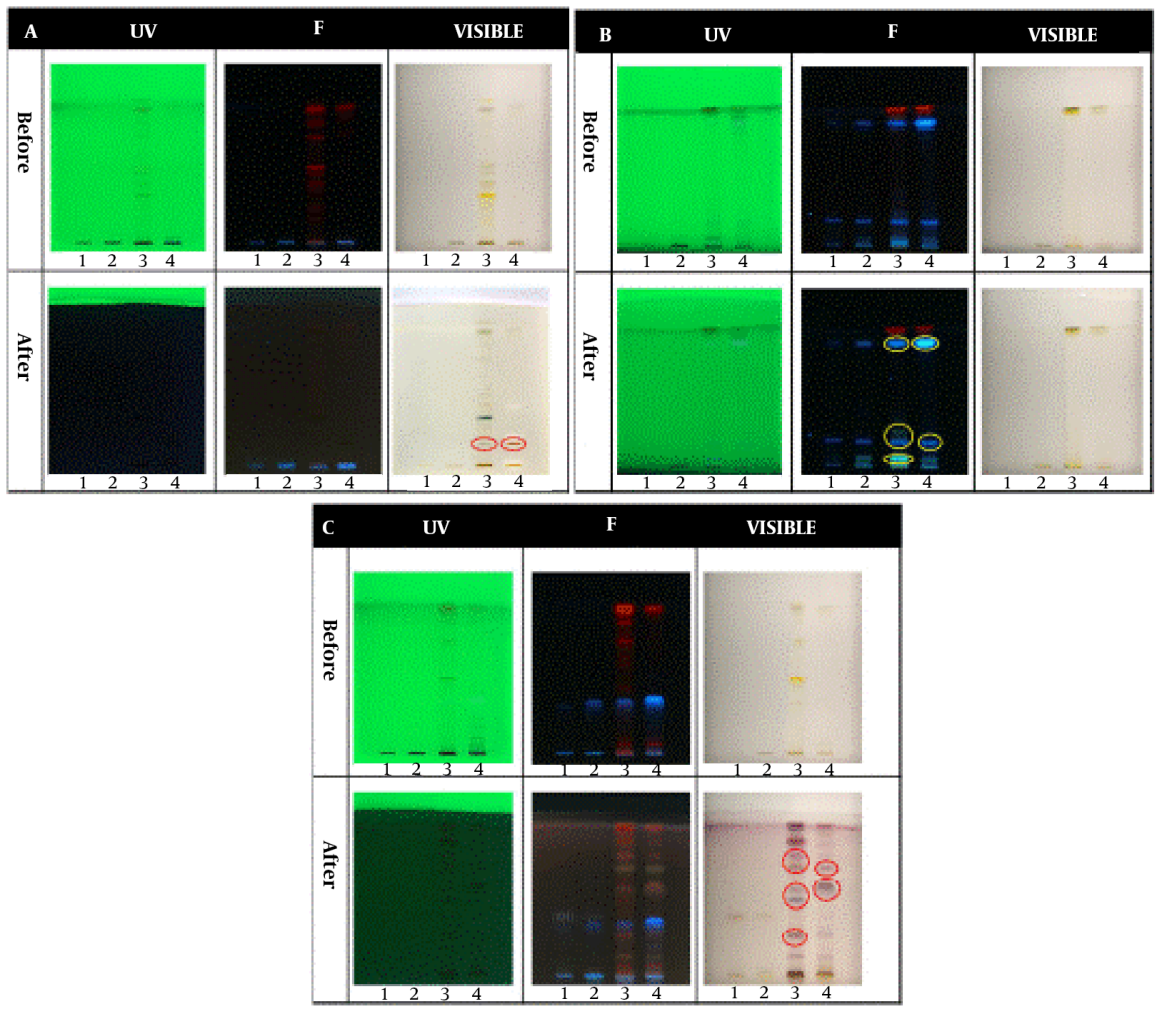
HPTLC results for identifying (A) alkaloids, (B) flavonoids, and (C) terpenoids. Spots 1, 2, 3, and 4 belong to root, stem, leaf, and fruit extracts, respectively.

**Figure 2.**
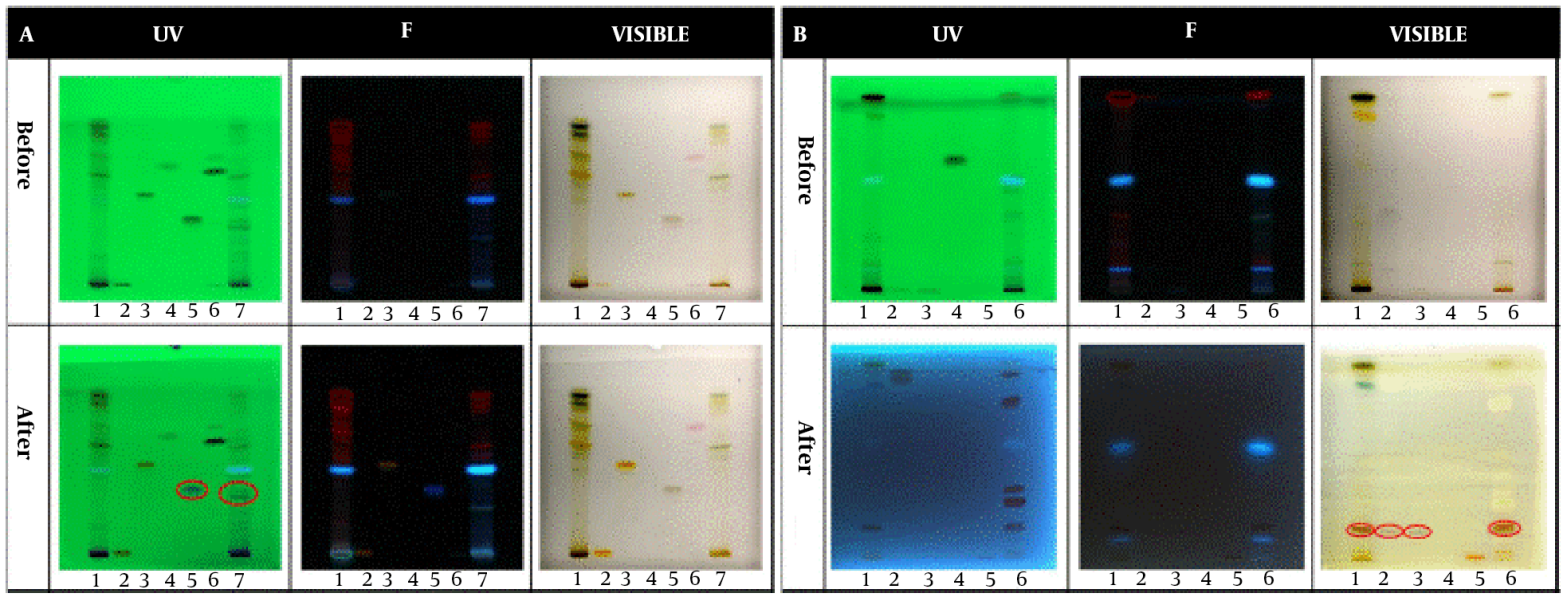
HPTLC results for specific identification of (A) Flavonoids and (B) Alkaloids. Spots 1, 2, 3, 4, 5, 6, and 7 in the flavonoid test belong to leaf extract, rutin standard, quercetin standard, benzoic acid standard, gallic acid standard, warfarin standard, and fruit extract, respectively. Spots 1, 2, 3, 4, 5, and 6 in the alkaloid test belong to leaf extract, belladonna standard, atropine standard, phenobarbital standard, hyoscine standard, and fruit extract, respectively.

In the qualitative tests, all TLCs were stained as follows; spot 1: Root extract, spot 2: Stem extract, spot 3: Leaf extract, and spot 4: Fruit extract. The results of the qualitative identification of alkaloids in different fractions of the *Atropa belladonna* plant are in Figure 1A. The stained TLC plates before and after derivatization were displayed under UV, fluorescence, and visible light. As shown, two brick-colored spots under visible light confirmed the presence of alkaloids in the leaf and fruit extracts after derivatization. A qualitative identification test for flavonoids is presented in Figure 1B. After the derivatization process, a color change was observed under fluorescence; these spots confirmed the presence of flavonoids in leaf and fruit extracts. As shown in Figure 1C, purple spots under visible light that appeared after derivatization confirmed the presence of terpenoids in leaf and fruit extracts. The results of all three qualitative tests for alkaloids, flavonoids, and terpenoids illustrated that the fruit and leaf of this plant contained these compounds. Hence, HPTLC was carried out to identify specific flavonoids and alkaloids in the fruit and leaf fractions using rutin, quercetin, gallic acid, and warfarin as flavonoid standards and belladonna, atropine, phenobarbital, and hyoscine as alkaloid standards. According to the ultraviolet results in Figure 2A, gallic acid was one of the main flavonoids in the fruit extract. The results of the specific alkaloids test showed two brick-colored spots under visible light (Figure 2B), confirming the presence of belladonna and atropine alkaloids in the leaf and fruit of the plant.

### 4.2. Determination of Total Alkaloid Content Using HPLC

Considering the desired initial weight of the extract for solubilization, the amount of atropine and scopolamine in the extract is measured by HPLC (8). The obtained chromatograms of scopolamine and atropine analysis are displayed in Appendix 2 in the Supplementary File, and the quantitative analysis results of these two alkaloids in different parts of the *Atropa belladonna* plant are given in Table 1. As can be seen in Table 1, the highest amount of scopolamine and atropine was found in the fruit extract (8.74 mg/g and 46.7 mg/g, respectively). Scopolamine was not detected in the root extract, and the stem extract contained the lowest amount of atropine (4.91 mg/g). It is worth mentioning that a significant amount of both scopolamine and atropine compounds was present in the leaf extract (7.54 mg/g and 38.74 mg/g, respectively).

**Table 1.** HPLC Quantitative Analysis Results of Atropine and Scopolamine in Fractional Extracts

Extract and Compound	Results, mg/g (No. (%))
**Root**	
Scopolamine	ND
Atropine	8.11 (0.811)
**Stem**	
Scopolamine	3.2 (0.32)
Atropine	4.91 (0.491)
**Leaf**	
Scopolamine	7.54 (0.754)
Atropine	38.74 (3.874)
**Fruit**	
Scopolamine	8.74 (0.874)
Atropine	46.7 (4.67)

### 4.3. Determination of Volatile Components Using Gas Chromatography-Mass Spectrometry

Gas Chromatography-Mass Spectrometry was performed to identify and quantify the volatile components in the fractional extracts. According to the results presented in Table 2, 5 different compounds were identified in the root extract, with diacetone alcohol (83.372%) as the major component. Moreover, some fatty acids such as oleic acid (8.536%), palmitic acid (3.925%), and stearic acid (3.05%) were detected in the root extract. Volatile components in the stem extract are presented in Table 2. The most abundant volatile compounds in the stem extract were mesityl oxide (47.276%) and oleic acid (26.035%). Stearic acid and palmitic acid were identified in the stem extract at 13.581% and 8.408%, respectively. In the leaf extract, 14 volatile compounds were detected, with palmitic acid (23.3%), *cis*-vaccenic acid (19.38%), atropine (15.01%), and stearic acid (11.66%) as the major components. Seven volatile compounds in the fruit extract are displayed in Table 2. Linoleic acid (40.79%), diacetone alcohol (24.93%), and capric acid (11.749%) were the major volatile components recognized in the extract.

**Table 2.** Gas Chromatography-Mass Spectrometry Qualitative and Quantitative Results of Fractional Extracts

Extract and Compound	Retention Time, min	Area, %
**Root**		
2-Pentanone, 4-hydroxy-methyl (Diacetone alcohol)	6.913	83.372
9-Eicosyne	21.819	1.117
n-Hexadecanoic acid (Palmitic acid)	23.253	3.925
Oleic acid	24.943	8.536
Octadecanoic acid (Stearic acid)	25.12	3.05
Total	-	100
**Stem**		
3-Penten-2-one, 4-methyl (Mesityl oxide)	5.37	47.276
2-Pentanon, 4-hydroxy-4-methyl (Diacetone alcohol)	6.884	2.78
2-Pentanon, 4-methoxy-4-methyl	8.486	1.919
n-Hexadecanoic acid (Palmitic acid)	23.261	8.408
Oleic acid	24.958	26.035
Octadecanoic acid (Stearic acid)	25.143	13.581
Total	-	99.991
**Leaf**		
Propanedioyl dihydrazide (Malonic dihydrazide)	5.226	6.716
3,7,11,15-tetramethyl-2-Hexadecen-1-ol acid	24.515	1.891
Pentadecanoic acid, 14-methyl-, methyl ester	25.072	1.088
n-Hexadecanoic acid (Palmitic acid)	25.772	23.3
7-Octadecenoic acid, methyl ester	27.188	2.761
Phytol	27.376	2.424
Octadecenoic acid, methyl ester	27.474	1.255
*cis*-Vaccenic acid	27.865	19.386
Octadecenoic acid (Stearic acid)	28.099	11.665
Atropine	28.957	15.01
Hexadecanoic acid, 1-(hydroxymethyl) -1,2-ethanediyl ester (1,2-Dipalmitoyl-rac-glycerol)	29.364	5.424
*cis*-11-Eicosenamide	30.199	2.854
Glycidol stearate	31.449	3.477
Total	-	100
**Fruit**		
2-pentanon, 4-hydroxy-4-methyl (diacetone alcohol)	6.848	24.939
2-pentanon, 4-methoxy-4-methyl	8.501	1.450
n-decanoice acid (capric acid)	16.773	11.749
n-hexadecanoic acid (palmitic acid)	23.254	8.120
9,12-octadecenoic acid, methyl ester,(E,E)-( linolelaidic acid )	24.365	4.201
9,12-octadecenoic acid, methyl ester,(Z,Z)-( linoleic acid )	24.922	40.794
β –D-glucopyranose, 1,6-anhydro- (levoglucosan)	25.112	8.746
Total	-	99.99

### 4.4. Nanofiber Preparation

To explore the advantages of nanofibers, the plant extract was employed to produce the mat using the electrospinning method. According to the antibacterial test, the fruit of *Atropa belladonna* was known for its exceptional antibacterial properties among other fractional extracts. However, there was limited availability of fruit extract for producing a variety of nanofiber formulations. As an alternative, the leaf extract, which showed a slightly lower antibacterial activity, could have been used. In this study, 40 nanofiber formulations of leaf extract were produced. Microscopic investigations disclosed that 5 nanofibers out of 40 formulations had a proper structure for further analysis using SEM, contact angle measurements, and tensile and antibacterial tests.

### 4.5. SEM Analysis

**Figure 3.**
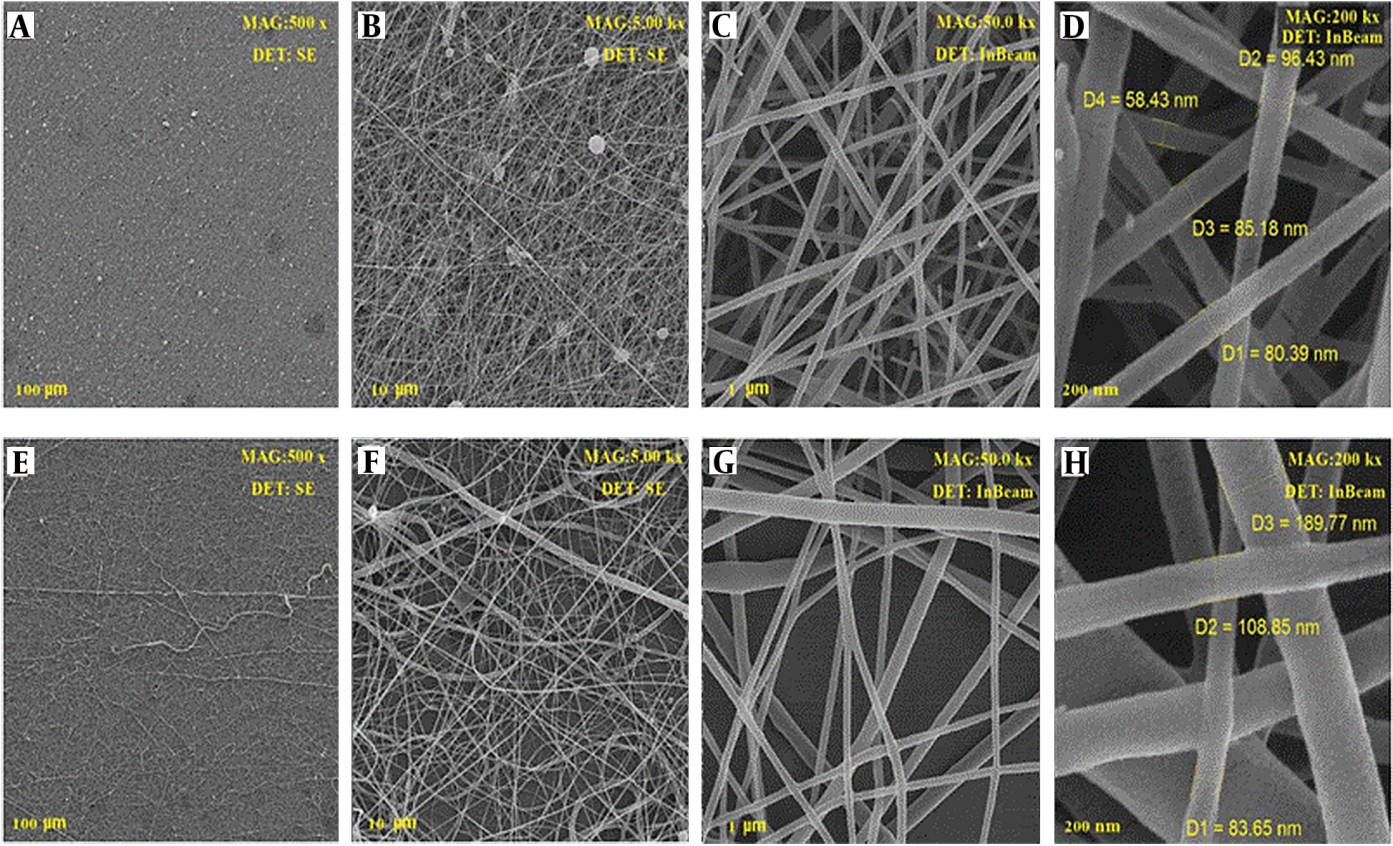
SEM images of PCB (PEO: Chitosan)

**Figure 4.**
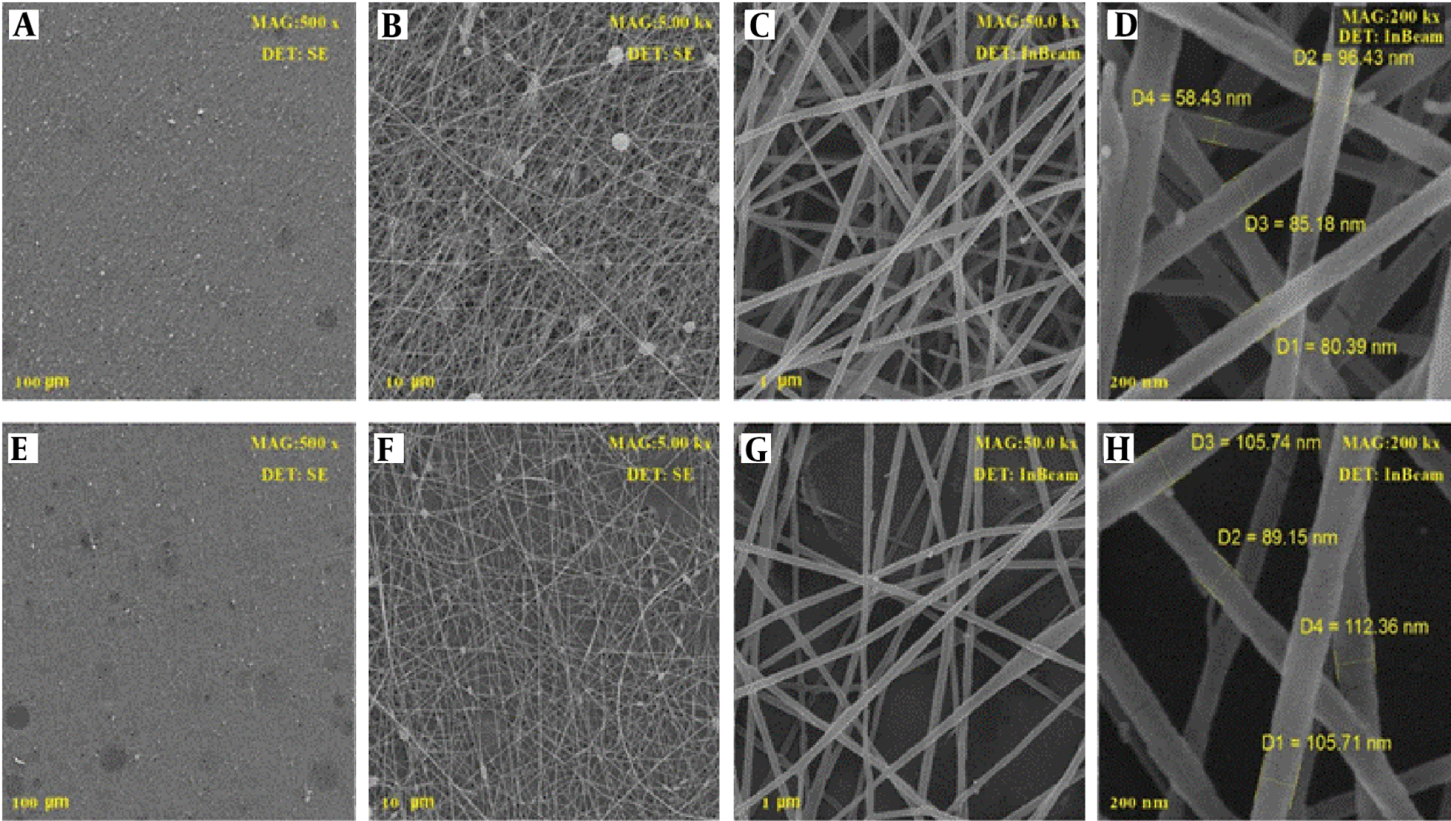
SEM images of PCE (PEO: Chitosan with extract)

To find out the morphological parameters of produced nanofibers, scanning their surface was carried out using an SEM device. The nanofibers were encoded as PCL 20% (PC20), PEO: Chitosan as Blank (PCB), PCL/PEO: Chitosan as Blank (PPCB), PEO: Chitosan with extract (PCE), and PCL/PEO: Chitosan with extract (PPCE). The SEM images of the PCB are displayed in Figure 3. All the images were taken with two detectors at different magnifications from different points of the nanofibers. Clearly, the PCL polymer with a weight percentage of 20%, which also serves as a scaffold in the context of the photos containing PCL, exhibited a suitable and uniform morphology with a mean diameter of 180 nm. The structure of the mixture of PEO and chitosan at a ratio of 80:20 with diameters of 80 - 135 nm is displayed in Figure 3A-D. In this image, the fiber has not yet been loaded with the extract. Figure 4A-H displays the SEM images of the mixture of PEO and chitosan at a ratio of 80:20 loaded by plant extract. The images show good morphology with a diameter of 90 - 112 nm. Moreover, it can be seen that after adding the leaf extract, the morphology of nanofibers remained unchanged. However, the extract altered the order of single polymer chains and decreased the solution viscosity (14). The images show that the fiber had very good uniformity and morphology. This intact morphology was caused by the low polarity of the extract, which did not impact the conductivity of the polymer solution (14).

### 4.6. Contact Angle Test

**Figure 5.**
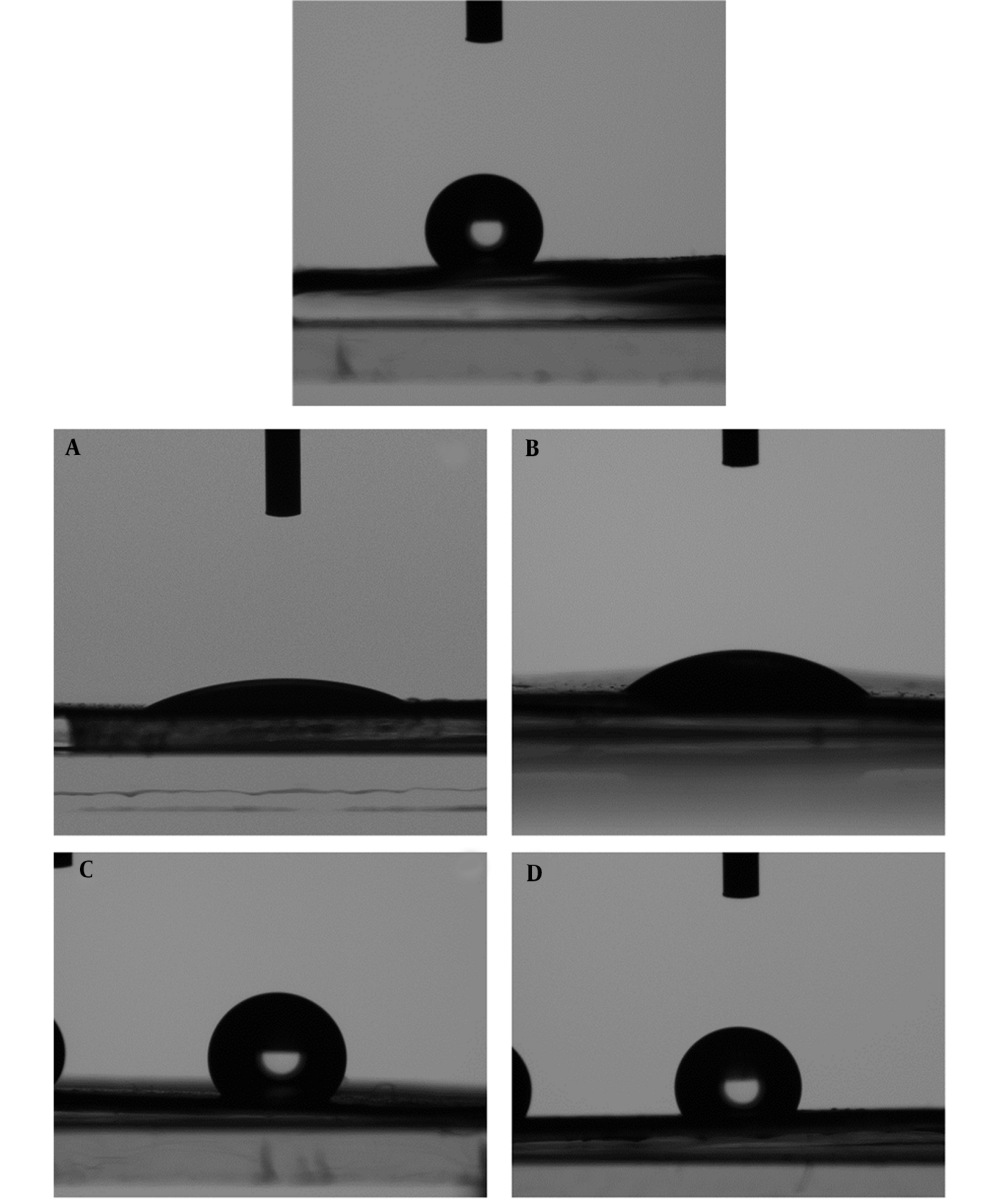
Water drop on PC20 (Top), PCB (A), PPCB (B), PCE (C), and PPCE (D)

The contact angle measurement was recorded with a CCD camera to check the hydrophobicity, hydrophilicity, and wettability of the produced mat. The images are shown in Figure 5. According to the principles of this test, substances with a water contact angle ranging from 0 to 90 are categorized as hydrophilic, while those with water contact angles greater than 90 are classified as hydrophobic or lipophilic. The contact angle results from the right and left sides of the water drop in contact with the surface of the samples are presented in Table 3. The findings show that the nanofibers in both PCB and PCE samples, which comprise a mixture of PEO and chitosan, are hydrophilic, irrespective of the existence of the plant extract. After loading the extract on the PCB fiber, the contact angle slightly increased, implying that the hydrophilicity decreased in the presence of plant extract. It can be attributed to the extract's low polarity. The tests conducted on PCL20, PPCB, and PPCE samples demonstrated the identical lipophilic characteristics of the fabricated nanofibers. This can be attributed to the surface roughness. A rough surface reduces the contact angle by providing more area for the liquid to adhere to. The opposite is true, i.e., a smoother surface can increase the contact angle.

**Table 3.** The Contact Angle from the Right and Left Sides of the Water Drop on the Surface of Nanofiber Formulations

Formulation	Test Items							
	L_1_ (°)	R_1_ (°)	L_2_ (°)	R_2_ (°)	L_3_ (°)	R_3_ (°)	L_Ave_ (°)	R_Ave_ (°)
**PCL20**	120	130	130	127	124	126	125	128
**PCB**	24	21	27	27	25	25	26	24
**PCE**	35	31	44	40	40	42	40	36
**PPCB**	126	128	120	130	123	132	123	130
**PPCE**	124	117	120	119	110	105	122	118

Abbreviations: L, left angle; R, right angle.

### 4.7. Tensile Test

**Figure 6.**
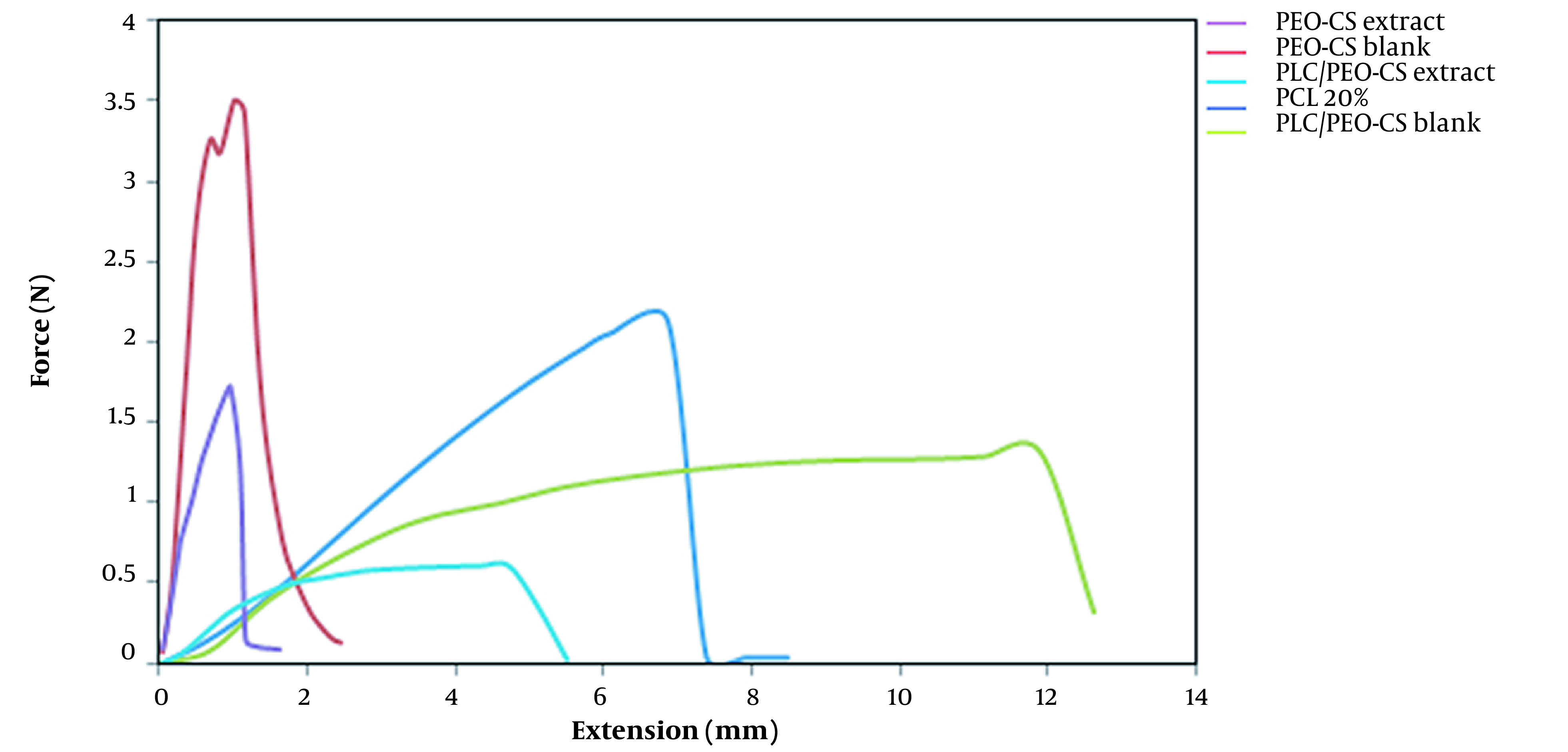
Tensile test graphs of 5 nanofiber formulations

The tensile test assessed the mechanical properties of the 5 selected formulations. The tensile test is a mechanical test using a gradually increasing force on the specimen until it breaks. The graphs obtained from the tensile test are presented in Figure 6. Generally, polymeric materials undergo a two-step process when stretched. First, they deform elastically, meaning they revert to their original shape after releasing the tension. Second, with increasing stress, they undergo plastic deformation and finally tear. According to diagrams obtained from the tensile test of 5 different formulations, it was found that PCL, PCB, and PCE nanofibers broke and tore suddenly, whereas the PPCB and PPCE formulations entered the elastic part at first and then were plastically deformed. Finally, they broke (tore), suggesting that these formulations are stronger and more appropriate. Table 4 shows the value of ultimate tensile strength, Young's modulus, and elongation. The ultimate tensile strength of PCB and PPCB was 3.5 and 1.32, which decreased to 1.68 and 0.61 when the extract was added to them. On the other hand, like the ultimate tensile strength, the elongation of neat fibers was reduced when the extract was embedded in the fibers. This can be elucidated by the fact that adding the extract to the nanofibers reduced the solution's viscosity, causing a decrease in the fiber diameter and consequently lowering the fibers' mechanical properties (15).

**Table 4.** Tensile Test of Nanofiber Formulations

Formulation	Test Items			
	Thickness, mm	Width, mm	Gage Length, mm	Test Speed, mm/min
**PCL20**	0.039	9	23.6	5
**PCB**	0.117	9.15	12.82	5
**PCE**	0.057	8.68	16.15	5
**PPCB**	0.042	9	25.9	5
**PPCE**	0.028	11.51	29.4	5

### 4.8. Antioxidant Activity

The DPPH free radical scavenging activity test determined the fraction's antioxidant activity. Ascorbic acid was used as a reference standard and showed radical scavenging activity with an IC_50_ value of 8600 ± 0.1 µg/mL. In this assay, leaf extract showed the highest ability for radical scavenging activity (IC_50_ = 135.68 ± 0.05 µg/mL). Other fractions obtained from root, stem, and fruit have relatively weak antioxidant properties (Table 5). The leaf extract had a greater concentration of herbal alcohols and fatty acids than other extracts, likely due to its high antioxidant activity.

**Table 5.** Results of the DPPH Test

Extract	Results (IC_50_, µg/mL)	Standard	Results (IC_50_, µg/mL)
**Leaf**	135.68 ± 0.05	Ascorbic acid	8.6 ± 0.1
**Root**	600.19 ± 0.18		
**Stem**	802.30 ± 0.19		
**Fruit**	943.21 ± 0.35		

### 4.9. Antimicrobial Activity of Fractional Extracts

Paper filter disks impregnated with the tested extract fraction in agar wells were used to assess the qualitative antimicrobial test against four strains of *Pseudomonas aeruginosa*. The diameters of microbial growth inhibition zones around the filter disks were measured, as presented in Table 6. The quantitative MIC and MBC test results are shown in Tables 7 and 8, respectively. Accordingly, the fractions obtained from the root, leaf, and fruit showed excellent antibacterial effects. Among fractional extracts, the total fruit extract possessed the highest antibacterial activity, and the total leaf extract showed potent antibacterial properties.

**Table 6.** Zone of Inhibition Test Results (mm)

Extract	Strain				
	STD9027	1	2	3	4
**Stem**	14	17	16	15	16
**Root **	19	20	18	16	18
**Leaf **	30	31	32	33	30

**Table 7.** Results of Minimum Inhibitory Concentration (MIC) Test (mg/mL)

Extract	Strain				
	STD9027	1	2	3	4
**Stem **	62.5	62.5	62.5	31.25	62.5
**Root**	15.62	15.62	31.25	31.25	15.62
**Leaf**	3.90	1.95	1.95	3.90	0.97
**Fruit **	0.00625	0.0125	0.00625	0.0031	0.00625

**Table 8.** Results of Minimum Bactericidal Concentration (MBC) Test (mg/mL)

Extract	Strain				
	STD9027	1	2	3	4
**Stem **	9.92	9.92	9.92	9.92	9.22
**Root**	42.68	21.34	21.34	21.34	21.34
**Leaf**	12.5	12.5	12.5	12.5	12.5
**Fruit **	6.25	6.25	6.25	6.25	6.25

### 4.10. Antibacterial Test of the Final Nanofiber Formulation

**Figure 7.**
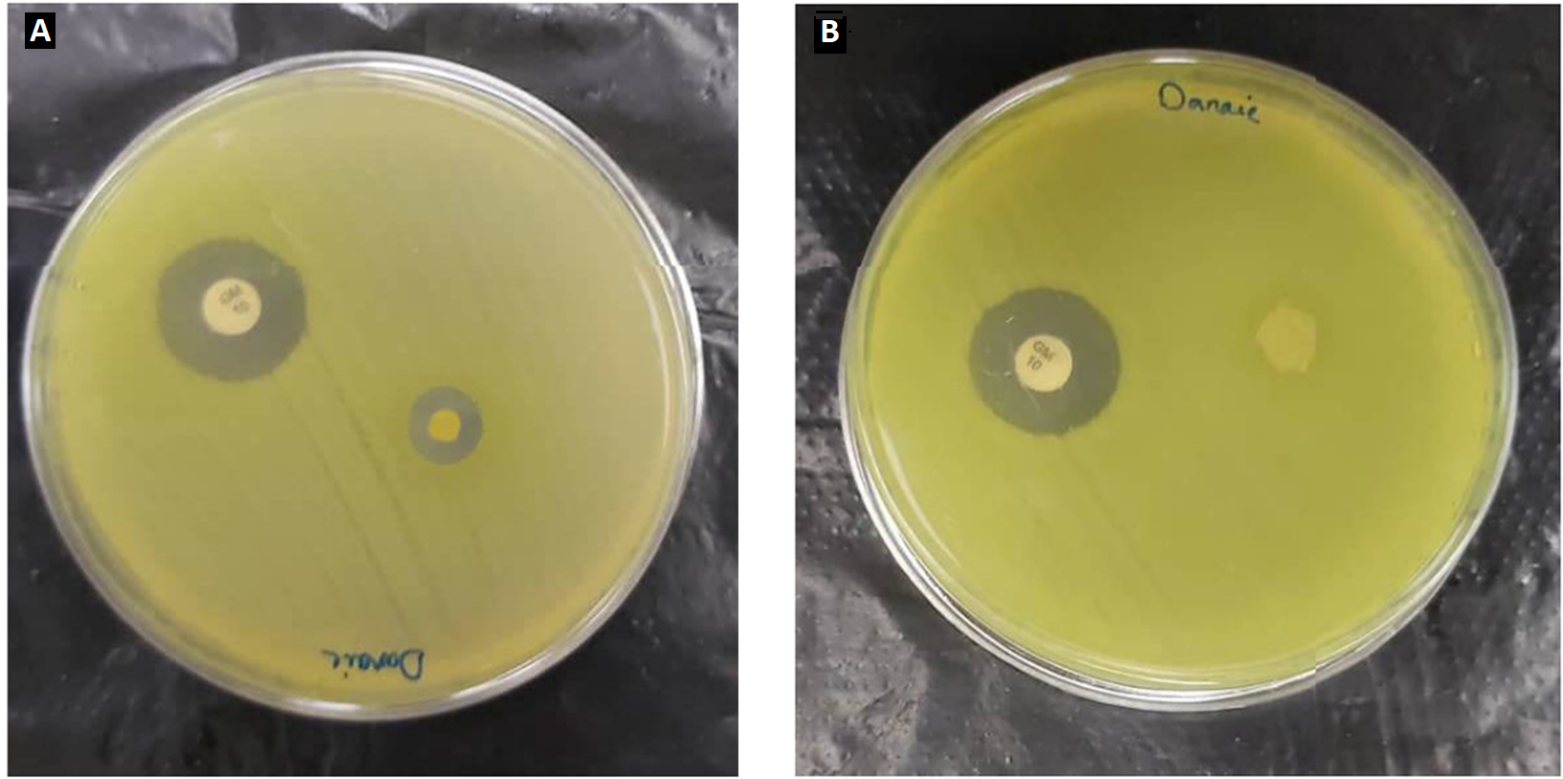
Disk diffusion test of extract-loaded (A) and extract-free PCL/PCO (B) nanofiber formulations against *Pseudomonas aeruginosa*. Gentamicin was used in both tests as a positive control.

A *Pseudomonas aeruginosa* bacterial suspension was obtained from the hospital to carry out the disk diffusion test. The suspension was prepared to a specific concentration of 0.5 McFarland equal to 10^8^ CFU/mL. The prepared suspension was cultured on Mueller-Hinton agar medium. The procedure was followed by placing the mat samples on the culture medium and incubating them at 37°C for 24 hours. As shown in Figure 7, PCL/PEO-CS formulation without extract exhibited no inhabitation zone. However, all three final nanofiber samples displayed inhibition zones of 8.4, 9, and 8 mm, with an average of 8.5 mm, thereby confirming the presence of antibacterial properties in PPCE upon loading the leaf extract on the nanofiber. This result can be explained based on the presence of the most well-known component of the plant, atropine, which has been shown to have antimicrobial activity against various bacteria, including gram-negative strains and fungi (16).

Other alkaloids present in *Atropa belladonna*, such as hyoscyamine and scopolamine, have also been found to have antibacterial properties. Alkaloids' antimicrobial activities result from their ability to interfere with various cellular processes of microorganisms, including DNA replication, protein synthesis, and cell wall synthesis. In addition, fatty acids in the leaf extract loaded in the nanofiber structure, such as oleic, palmitic, stearic, linoleic, and linolenic acids, possess antifungal and antibacterial activities (17, 18). According to the GC-MS results in Table 2, the main components of the leaf extract are cis-vaccenic acid, atropine, and octadecanoic acid, which exist with a high concentration in the extract. Therefore, it can be expected that the antibacterial effect of PPCE might also be due to the presence of fatty acids and atropine in the leaf extract.

The general mechanisms proposed for the antibacterial effects of fatty acids include disrupting bacterial cell membranes, altering cell membrane fluidity and permeability, causing leakage of the bacterial cytoplasm, and ultimately cell death. As another mechanism, fatty acids can interfere with intracellular processes, such as energy production or protein synthesis, by disrupting or inhibiting specific cellular enzymes and pathways. The combination of alkaloids and fatty acids present in the leaf extract of *Atropa belladonna* exhibits a synergistic effect, which leads to the inhibition of bacterial growth and proliferation. These mechanisms work together to prevent the spread and survival of bacterial pathogens, making the *Atropa belladonna* extract a potent natural antibacterial agent. The findings validate the possibility of using the PPCE formulation of nanofibers containing *Atropa belladonna* plant extract as an efficient infection treatment.

### 4.11. Conclusions

The characteristics of *Atropa belladonna* plant extract were studied in detail and used to prepare antibacterial nanofibers. The antioxidant study of the extract with the DPPH method confirmed that antioxidant compounds are abundantly found in the leaves of this plant. The HPTLC test showed the presence of gallic acid flavonoid, atropine, belladonna alkaloid, and terpenoids in the fruit and leaf extracts of this plant. The HPLC results showed that scopolamine was not found in the water extract of the root, and the fruit extract accounted for the largest amount, 0.874%. Likewise, the composition of atropine in the fruit had the highest amount, equal to 4.67%. The results of GC-MS analysis confirmed that the desired extracts contained significant amounts of fatty acids such as stearic acid, vaccenic acid, and oleic acid. Different antibacterial tests showed that the total extract of the fruit and leaves had very good antibacterial properties against *Pseudomonas aeruginosa*. In the production of electrospun nanofibers using plant leaf extract, the formulation that included PCL scaffold and chitosan-PEO exhibited a highly uniform and smooth surface, as observed in the SEM analysis. Contact angle measurement showed an angle between 90° and 180° that confirmed the relative hydrophobicity or lipophilicity; hence, it is expected that the extract is released from the mat in a controlled manner to exert its antibacterial effect on the wound. The leaf extractive-loaded nanofiber also showed good antibacterial properties with a mean inhabitation zone of 8.5 mm. These results confirm the potential of this formulation as an effective one for further research. It suggests the use of *Atropa belladonna* plant extract in the treatment of infections, specifically in the form of nanofibers. Additionally, it proposes the utilization of this extract in hospital wound bandages.

ijpr-22-1-137839-s001.pdf
