# Supplementary material for: Chemical Composition Analysis of Atropa belladonna Grown in Iran and Evaluation of Antibacterial Properties of Extract-loaded Nanofibers
Source: Iran J Pharm Res. 2023 Aug 6;22(1):e137839. doi: 10.5812/ijpr-137839 (PMC10750788; doi:10.5812/ijpr-137839)
Supplement: ijpr-22-1-137839-s001.pdf [file ijpr-22-1-137839-s001.pdf]

Chemical Composition Analysis of Atropa Belladonna Grown in Iran and Evaluation of Antibacterial Potential of Extract Loaded Nanofibers

Appendix 1: Amount of solid extract obtained from Atropa belladonna plant.

Fraction name	Aqueous-R	Chloroform-R	Aqueous-S	Chloroform-S	Aqueous-L	Chloroform-L
Amount (g)	5.55	0.34	3.48	0.19	4.72	1.24

R:root, S: stem, L: leaf

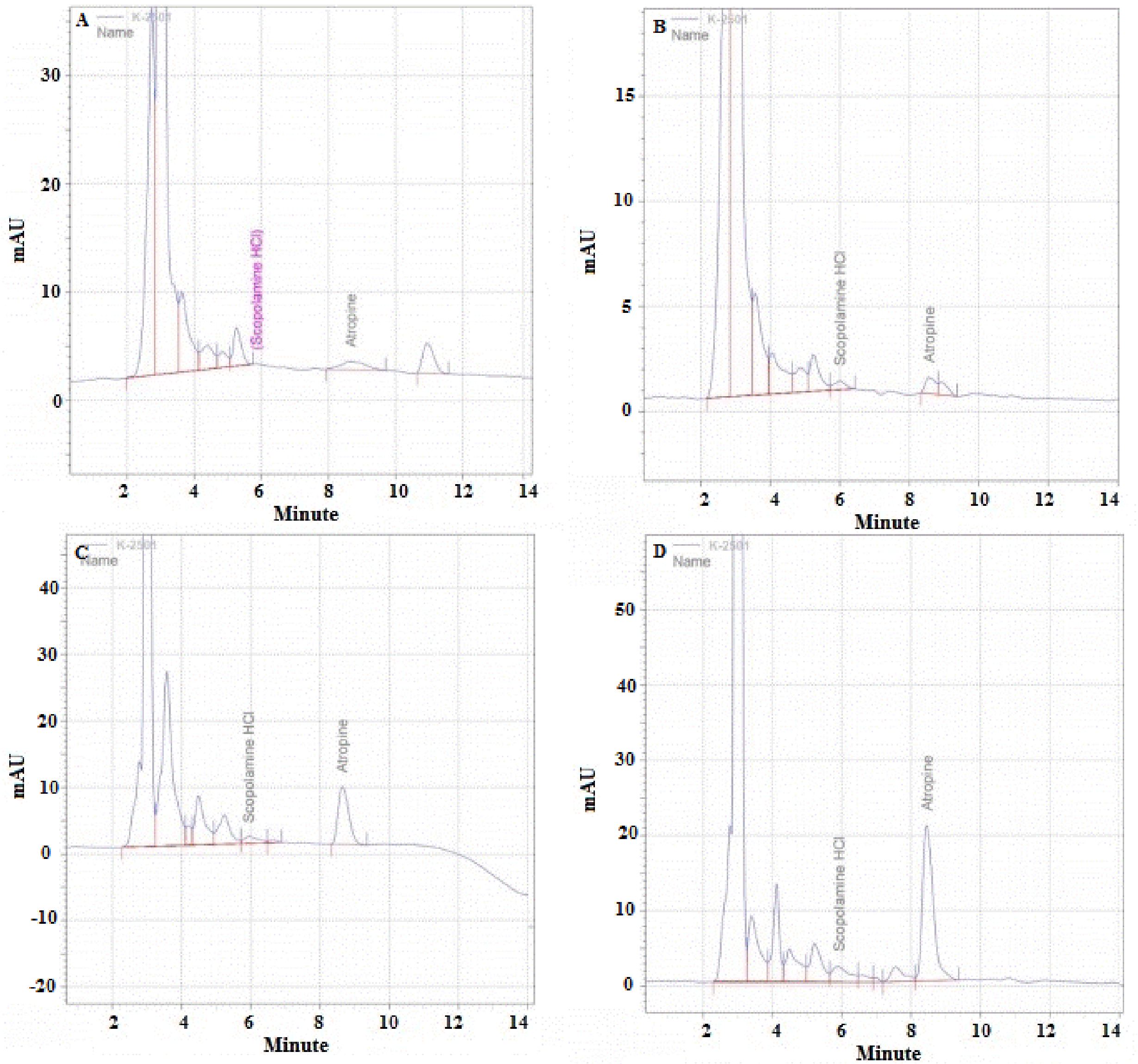

Appendix 2. HPLC chromatograms of Scopolamine and Atropine

Appendix 3: Mechanical properties of electrospun nanofiber formulations

Formulation	Test Items		
	**Ultimate force (N)**	**Young modulo**	**Elongation (%)**
PCL20	2.12	0.32	204
PCB	3.50	6.83	128
PCE	1.68	2.92	104
PPCB	1.32	3.84	210
PPCE	0.61	0.23	122

Preparation of 0.5 McFarland microbial suspensions

To prepare the microbial suspension, after the growth of the bacterial colony, the surface of the bacterial culture medium was removed and inoculated in the Mueller Hinton Broth medium to reach the required turbidity equal to 0.5 McFarland. The absorbance of this sample at a wavelength of 625 nm will be equal to 0.08-0.1 au, and this sample has a density of about 1.5 x 10^8 CFU/ml.

Dilute the extracts for the well test

One gram of Atropa belladonna stem, root, and leaf extracts and their aqueous fractions were weighed. Total extracts were diluted 1:1 with water once; as a result, 12 samples were prepared for the well test. The solvents and concentrations prepared are specified in Appendix 4.

Appendix 4: The employed solvents and prepared concentrations from *Atropa belladonna* extracts

Extract	Solvent	Solvent volume (mL)	Final concentration (mg/mL)
stem extract	Deionizer water	2	500
root extract	Deionizer water	2	500
leaf extract	DMSO	4	250

Well plate test

Mueller Hinton Agar medium is needed to perform this test. 20 to 21 microliters of molten (sterilized) culture medium were poured into 10 cm plates and given time to become solid. Microbial suspension with a concentration of 0.5 McFarland was spread on the culture medium with a Swab (cultivated). Using a well-making rod, wells were created on the surface of agar

according to the number of samples intended to measure their effectiveness. Then, the inside of the wells was filled with the desired material. The volume of the material that was poured into the wells was the same. The plates were kept in an incubator for 24 hours at a temperature of 37°C and the results were recorded.

Appendix 5: The characteristics of stationary and mobile phase as well as derivatization related to alkaloids, flavonoids, and terpenoids performed with HPTLC.

Test	Stationary Phase	Mobile Phase	Derivatization reagent
alkaloids	Silica gel 60 F 254 HPTLC Plate 10*10	Toluene-Ethyl acetate-Diethylamine (7:2:1)	Dragendorff ($Bi(NO_3)_3$), tartaric acid, and potassium iodide
Standard -alkaloids	Silica gel 60 F 254 HPTLC Plate 10*10	Acetone-water-ammonia (9:4:0.7:0.3)	dragendorff
flavonoids	Silica gel 60 F 254 HPTLC Plate 10*10	Toluene-Ethyl acetate-Formic acid-Acetic acid (10:1.1:1.1:2.6)	Natural products
Standard flavonoids	Silica gel 60 F 254 HPTLC Plate 10*10	Toluene-Ethyl acetate-Formic acid-Acetic acid (6:4:0.5:0.5)	Natural products
terpenoids	Silica gel 60 F 254 HPTLC Plate 10*10	Hexane-Ethyl acetate (1:1)	Anisaldehyde-sulfuric acid

Catalase test

To perform this test, some of the bacterial colony was dissolved in a drop of H_2O_2. If gas bubbles are seen, it means that the bacteria has catalase enzyme and can convert H_2O_2 into hydrogen gas and water (Appendix 6).

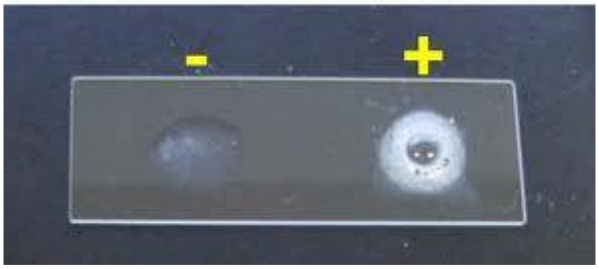

Appendix 6: result of catalase test

Oxidase test

This test is used to identify and classify Gram-negative bacteria. This test is an indirect test to determine the presence of the Cytochrome C oxidase enzyme. The substance that regenerates the oxidase reagent is Cytochrome C. So the positive result of the test shows the presence of Cytochrome C and these bacteria have this enzyme as a respiratory enzyme. This enzyme is the only soluble Cytochrome that is examined in the oxidase test. The reagent of this test is N, N, N′, N′-tetramethyl-p- phenylenediamine which is easily oxidized by Cytochrome C and produces purple color. This reagent is sensitive to light and oxidizes quickly, so it is stored in dark glass. To perform the test, dip a piece of filter paper or swab in the solution containing 1% N,N,N′, N′-tetramethyl-p- phenylenediamine, then transfer the colony of the tested bacteria to the paper or swab. If the test is positive, the colonies will turn purple, which indicates the presence of oxidase-positive bacteria. The result must be checked after 15 seconds; otherwise it will be worthless (Appendix 7).

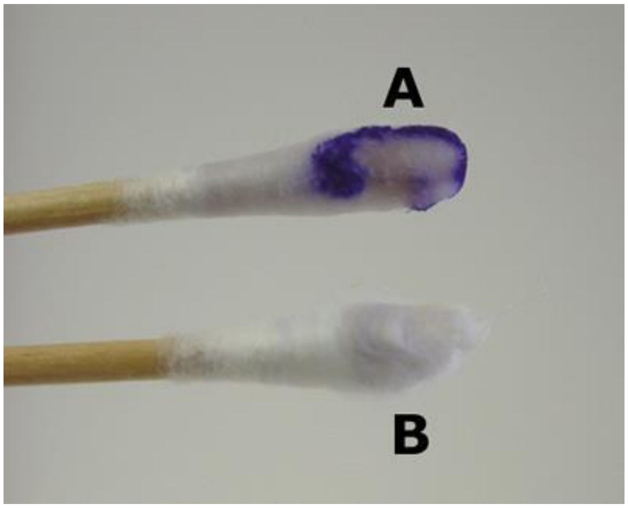

Appendix 7: result of oxidase test (A: positive, B: negative)

The results of Pseudomonas aeruginosa culture and its confirmation with biochemical and morphological tests

First, the isolated strains were cultured in Mueller Hinton agar medium, and then gram staining test was performed on all 5 strains, all of which showed gram-negative bacilli (Appendix 8).

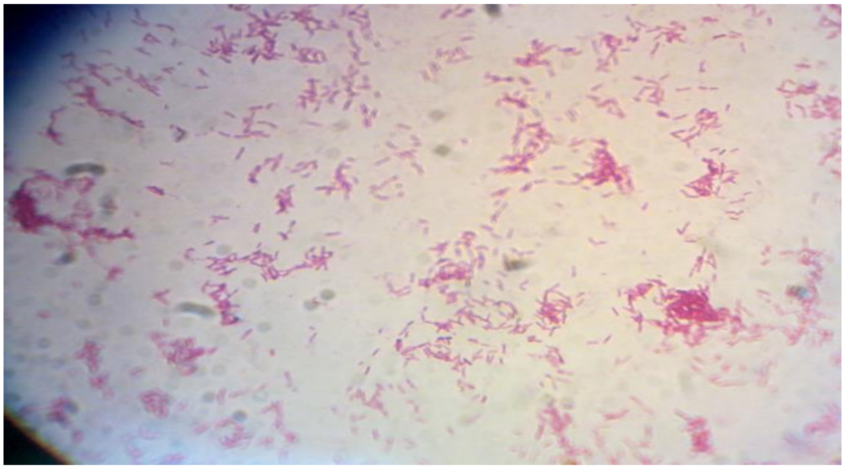

Appendix 8: Gram staining microscopic image of Pseudomonas aeruginosa isolated from patients hospitalized in Shahid Motahari Hospital, Tehran

The results of other Pseudomonas identification and confirmation tests, including Catalase and Oxidase tests, are shown in the Appendix 9, which confirmed that the isolated clinical strains were Pseudomonas.

Appendix 9: result of Pseudomonas identification and confirmation tests

Strain	O/F	Catalase	Oxidase	Coloring	Growth in 42°C	Arginine Dihydrolase
1	O^+	+	+	+	+	+
2	O^+	+	+	+	+	+
3	O^+	+	+	+	+	+
4	O^+	+	+	+	+	+

Antibiogram test results

To identify the resistance of the isolated strains, the Antibiogram test was performed using the antibiotic discs Polymyxin B 300 µg, Amikacin 30 µg, Tobramaycin 10 µg, Ciprofloxacin 5 µg, and Gentamicin 10 µg. The size of the inhabitation zones is recorded in Appendix 10.

Appendix 10: The size of the inhabitation zones of the isolated strains

Strain	Gentamicin	Ciprofloxacin	Tobramaycin	Amikacin	Polymyxin B
1	10mm	12mm	10mm	10mm	14mm
2	9mm	11mm	9mm	20mm	17mm
3	8mm	14mm	11mm	12mm	15mm
4	18mm	12mm	8mm	19mm	14mm

According to the diameter of the halos, it was determined whether the strains were resistant or sensitive according to the CLSI standard, the results of which are given in Appendix 11.

Appendix 11: resistant (R) or sensitive (S) behavior of the strains according to the CLSI standard

Strain	Gentamicin	Ciprofloxacin	Tobramaycin	Amikacin	Polymyxin B
1	R	R	R	R	S
2	R	R	R	S	S
3	R	R	R	R	S
4	S	R	R	S	S
